# Integrating lifestyle behaviors in school education: A proactive approach to preventive medicine

**DOI:** 10.1016/j.pmedr.2025.102999

**Published:** 2025-02-05

**Authors:** Mohamad Motevalli, Fatima Cody Stanford, Günther Apflauer, Katharina Christina Wirnitzer

**Affiliations:** a Department of Sport Science, University of Innsbruck, Innsbruck, Austria; b Department of Secondary Education, University College of Teacher Education Tyrol, Innsbruck, Austria; c MGH Weight Center, Department of Medicine-Division of Endocrinology-Neuroendocrine, Department of Pediatrics-Division of Endocrinology, Nutrition Obesity Research Center at Harvard (NORCH), Massachusetts General Hospital, Harvard Medical School, Boston, MA, USA; d Federal Ministry for Education, Science and Research, Department of School and University Sports, Vienna, Austria; e Research Center Medical Humanities, University of Innsbruck, Innsbruck, Austria; f Department of Pediatric Oncology and Hematology, Otto-Heubner Centre for Paediatric and Adolescent Medicine (OHC), Charité – Universitätsmedizin Berlin, Berlin, Germany

**Keywords:** Lifestyle, Education, Prevention, Health behavior, Public health, Health policy, Children

## Abstract

**Objective:**

Individual behavior is regarded as the primary factor influencing health. Despite recent advances in health science, the increasing prevalence of both non-communicable diseases and unhealthy lifestyle behaviors needs to be effectively controlled. The objective of this short communication is to explore the theoretical basis for integrating lifestyle habits into school education and highlight its significance for public health policy.

**Methods:**

A conceptual, perspective-driven analysis was conducted, drawing on evidence from existing literature on the role of educational settings in promoting healthy lifestyle behaviors. The study evaluates current physical education (PE) programs and curricula addressing health-related issues while proposing an updated, proactive approach to mitigating existing health concerns.

**Results:**

Schools have significant potential to cultivate healthy lifestyle habits through practical, real-life applications. PE is a primary school-based subject capable of promoting an active lifestyle; however, debates persist regarding its adequacy and effectiveness in other facets of health promotion. From a holistic perspective, it is proposed that PE be evolved into lifestyle education (LE), that aims to integrate all lifestyle factors—beyond physical activity—within the school-based educational framework on a global scale.

**Conclusions:**

Establishing school-based LE appears crucial for addressing the growing pandemic of chronic health conditions from their earliest origins.

## 1 Background

Individual behavior is considered the most significant determinant of health. Unhealthy behaviors, mainly including physical inactivity, poor diet, and substance abuse, have been known as the primary drivers of current worldwide health concerns (World Health Organization (WHO), 2022). These behaviors play a significant role in the prevalence of non-communicable diseases (NCDs), including cardiovascular diseases, cancer, metabolic disorders, musculoskeletal dysfunctions, and mental health problems, contributing to 74 % of all deaths globally (World Health Organization (WHO), 2022). Overweight/obesity, in particular, has emerged as an essential driver of these global health challenges, further intensifying the burden of NCDs (GBD, 2021). Despite recent advances in preventive health science, data show that the growing prevalence of unhealthy habits and the associated chronic conditions, including obesity, remains partially uncontrolled (GBD, 2021; Hruby and Hu, 2015).

Children and adolescents are vulnerable populations to health and behavioral problems. It has been well-established that unhealthy lifestyle behaviors often emerge and solidify during childhood/adolescence, persisting into adulthood (World Health Organization (WHO), 2008). Technological advances in the modern era, coupled with the associated inactive lifestyle, have partially influenced the phenotype of the current generation of children, resulting in higher body weight and body mass index (BMI) compared to their peers from former generations (Hruby and Hu, 2015). The prevalence of overweight and obesity among children and adolescents has risen dramatically from 8 % in 1990 to 20 % in 2022 (World Health Organization (WHO), 2024). Data from large-scale international studies indicate that the majority of children and adolescents follow unhealthy or poor physical activity (PA) and dietary behaviors (Centers for Disease Control and Prevention (CDC), 2022; World Health Organization (WHO), 2021a).

Lifestyle medicine (LM) is an evidence-based specialized field emphasizing health-related behaviors as the primary approach to preventing and treating chronic diseases (American College of Lifestyle Medicine ACLM, 2025). LM is based on the six components of lifestyle behaviors, including PA, diet, substances, sleep, stress, and relationships, collectively forming an interconnected, holistic framework for preventing and managing a wide range of health conditions (American College of Lifestyle Medicine ACLM, 2025). However, the type and significance of lifestyle factors and their impact on health may vary between different populations. For instance, “screen time” is also a critical health-related concern for today's children and adolescents, qualifying as a lifestyle component. Moreover, the increasing prevalence of vaping and e-cigarettes has emerged as a significant concern for adolescents, highlighting the need to broaden the focus beyond traditional tobacco and alcohol consumption (Nath et al., 2022). The interconnectedness between health behaviors suggests that lifestyle components naturally integrate to generate the most effective health promotion. Over the last few decades, a growing body of research has explored the independent and combined impacts of lifestyle components on health parameters; the data consistently indicate that multi-component interventions generally yield better outcomes than single-item approaches among various populations, including children and adolescents (Prochaska and Prochaska, 2011). Particularly, while each lifestyle component plays an independent role in the etiology and management of NCDs, combining lifestyle components (e.g., PA and diet) produces additional cumulative and synergistic results. Considering the greater health advantages of combining lifestyle elements, prioritizing a holistic health-promoting framework appears crucial for the current generation of children and adolescents.

## 2 Health and education

The confluence of health and education appears as a pivotal focus for the future, aligning with the United Nations Sustainable Development Goals (SDGs), particularly #3, “Good Health and Well-being,” and #4, “Quality Education”. According to the United Nations Educational, Scientific and Cultural Organization (UNESCO), “*Education is both a goal in itself and a means for attaining all the other SDGs*”. Considering that children and adolescents spend a substantial part of their waking time and daily activity period in school, this setting holds significant importance in disseminating health-related knowledge and cultivating health-related habits. Data from 199 countries worldwide reveal that compulsory school education spans five to 17 years, with an average of 10 years (World Bank Group, 2023). Therefore, since fostering healthy lifestyle habits requires prolonged time and continuous engagement, schools are ideal for shaping lifelong health-related behaviors. The World Health Organization (WHO) consistently advocates for school and educational policies prioritizing adopting and maintaining healthy lifestyle behaviors to improve public health (World Health Organization (WHO), 2008). The recently reported associations between lifestyle factors and the current COVID-19 pandemic reinforce the urgency of greater emphasis on health education in the school setting (Colao et al., 2020).

A Cochrane review concluded that the WHO's Health-Promoting Schools framework has shown promise in improving some aspects of health in school-aged children and adolescents (Langford et al., 2014). Nevertheless, reports indicate that the existing literature displays considerable heterogeneity in design and methodology among lifestyle interventions targeting children and adolescents, with the majority failing to address the most crucial aspect of a healthy lifestyle, i.e., its sustained effectiveness (Brown and Summerbell, 2009; Wang et al., 2013). In addition, it has been reported that interventional studies addressing children's PA and dietary habits commonly exhibit a high risk of bias (Brown and Summerbell, 2009; Wang et al., 2013), potentially stemming from the complexity of lifestyle components and the unintegrated nature of current approaches.

Despite the significant importance of lifestyle behaviors among children and adolescents, only one lifestyle factor (i.e., PA) currently holds an authorized position in school-based education. Physical education (PE), which primarily emphasizes PA, sports, and exercise, is compulsory in many educational systems. While the importance of PE extends beyond its defined roles, it is currently the most relevant subject for addressing health-related topics in schools, and it plays a leading role in developing “health literacy,” a vital component of the overarching educational goals (World Health Organization (WHO), 2021b). PE's effectiveness in promoting optimal PA behaviors is well-established, but concerns persist regarding its sufficiency and efficacy in other aspects of health promotion. While the health-promoting PA recommendation of 60 min/day is nearly unattainable within the existing 45–90 min/week of PE session(s), research shows that PE is not widely considered a life-changing and impactful educational topic among school subjects, with open discussions often downplaying its significance and effectiveness in life (United Nations Educational, Scientific and Cultural Organization (UNESCO), 2014). As an etymological fact, the phrase ‘physical education’ may not accurately convey non-physical topics related to health behaviors. This may lead to potential misinterpretations among various groups, diminishing the importance of PE as a practical health promotion approach within the educational framework.

Historically, when PE was introduced as a compulsory school subject in the first half of the 19th century, chronic diseases appeared less widespread, and unhealthy lifestyle choices were not as prevalent as today. Therefore, considering the undeveloped state of health-related research when PE was established as a compulsory school subject, it is reasonably presumable that the comprehensive understanding of lifestyle's impact on health had not yet emerged. Thus, there seem to be predominantly time-related justifications about why PE was initially defined and developed as the primary health-related school subject, focusing solely on PA/sport without incorporating other lifestyle components. In general, the potential adequacy of current PE in effectively addressing the growing prevalence of unhealthy lifestyle behaviors among young generations seems questionable, implying the need for implementing a more health-oriented and practical alternative.

## 3 It is time to establish lifestyle education

From a holistic, practical, and potentially life-changing perspective, it is time to upgrade PE to its modernized version, “Lifestyle Education” (LE). As a comprehensive and contemporary alternative to PE, LE seamlessly integrates all aspects of health behavior, aiming to capture both the individual benefits and the cumulative impact on overall well-being. Based on the LE approach, children and adolescents can acquire a comprehensive package of health-related knowledge, skills, and competencies that extend beyond PA and encompass all lifestyle components, including “diet,” which solely covers many sub-topics such as basic knowledge about (un)healthy food choices, energy balance, eating habits, nutrients, appetite, cooking, meal planning, kitchen rules, grocery shopping, and the food industry. Regarding diet, healthy school meal programs play a key role in LE by providing balanced meals rich in fruits, vegetables, and whole grains (Barnes et al., 2021). These programs can potentially cultivate a sense of social responsibility by teaching students the value of sharing meals, reducing food waste, and supporting environmentally friendly practices. Additionally, since adolescence is a time of rapid physical and emotional change, comprehensive sexuality education can also be an integral part of school-based LE programs that equip adolescents with the knowledge, skills, and attitudes necessary to make informed decisions about their sexual health and well-being (United Nations Educational, Scientific and Cultural Organization (UNESCO), 2024). This aspect of education may cover a broad range of topics, including human development, relationships, body image, sexual rights, contraception, gender identity, societal norms, and the prevention of sexually transmitted infections. Implementing LE appears crucial before expecting children and adolescents to adhere to the existing health-related recommendations.

A healthy lifestyle supports overall well-being and is crucial in promoting children's social and cognitive growth and optimizing their academic performance (Jirout et al., 2019), which leads to an increased likelihood of their future occupational success and generates a virtuous cycle of health and wellness. However, lifestyle behaviors' multifaceted and multi-setting nature highlights the importance of leveraging LE beyond formal education, particularly within families, peer groups, and social communities. Therefore, providing practical resources for parents/caregivers, promoting a healthy home environment, and organizing community activities and peer mentoring programs are crucial. Recognizing and harnessing the potential of LE outside of formal education can foster a broader environment that cultivates a lively culture of well-being. LE can also enhance the health-related knowledge and competencies of diverse individuals, encompassing children, adolescents, and those indirectly involved (e.g., family members and school staff), fostering a culture of health and well-being within communities and contributing to improved public health outcomes.

The LE approach requires practical actions from pedagogical/didactical administrations to design and implement associated courses, modules, curricula, and training opportunities. To effectively implement LE, educational authorities and policymakers must engage in comprehensive demonstrations regarding its vision, mission, goals, roles, and detailed guidelines tailored to various school levels and student demographics, including factors like gender, BMI, (dis)abilities, and socioeconomic status. Regular monitoring and feedback mechanisms are essential for evaluating the impact of LE programs and ensuring continuous improvement in program delivery and effectiveness. This can be achieved through an organized system of ongoing assessment by educational authorities and school administrations. A comprehensive understanding of social determinants of health is also crucial, necessitating adaptability and inclusivity in LE programs.

Additionally, understanding teachers' vital role as educators, counsellors, and role models and providing them with the required support and basic to advanced training and education to enhance their qualifications and competencies will contribute to the overall success of LE programs. To effectively implement school-based LE, it is also imperative to foster collaboration between educational organizations and medical institutions. This partnership can potentially encourage knowledge exchange, facilitate curriculum development, and promote mentorship programs, thereby enhancing the overall impact of LE initiatives. Furthermore, cross-sector collaborations between educational authorities and health professionals (including private sector partners in nutrition, fitness, and technology) can offer valuable resources and practical applications. For instance, integrating e-learning platforms and gamified tools can enhance the engagement and accessibility of LE, aligning with the demands of today's dynamic educational environment.

School-based LE is envisioned to play a pivotal role in aiding future public health policies to control the increasing pandemic of NCDs from their earliest origins, thereby reducing the burden of health conditions at both the public and global levels. It empowers younger generations with the knowledge and skills to make informed lifestyle choices, ultimately contributing to healthy longevity. The LE approach can impact primary healthcare, particularly in low- and middle-income countries. Expanding services to include preventive and wellness-oriented care can help strengthen recent healthcare reforms in developing countries and reduce the burden on secondary and tertiary care systems. Beyond its health benefits, LE is a sustainable strategy by addressing lifestyle-related environmental concerns, such as climate change.

## 4 Conclusion

The available epidemiological data and recent discoveries about the nature-nurture association in the etiology of NCDs provide robust rationales to expand the applied knowledge of healthy lifestyle behaviors. The primary step toward achieving this goal is setting up LE as a compulsory school subject, enabling children to acquire everyday knowledge and competencies concerning health-related behaviors within a curriculum-based educational framework. LE, which aligns with the widely accepted consensus that disease prevention outweighs medical treatment, empowers children and adolescents to adopt healthier lifestyles while fostering social and cognitive growth and contributing to healthy longevity. LE can help mitigate the rise of NCDs, lessening the public health burden and contributing to sustainable efforts addressing environmental concerns.

## Funding

This research received no direct funding.

## CRediT authorship contribution statement

**Mohamad Motevalli:** Writing – review & editing, Writing – original draft, Investigation, Conceptualization. **Fatima Cody Stanford:** Writing – review & editing. **Günther Apflauer:** Writing – review & editing. **Katharina Christina Wirnitzer:** Writing – review & editing, Investigation, Conceptualization.

## Declaration of competing interest

The authors declare that they have no known competing financial interests or personal relationships that could have appeared to influence the work reported in this paper.

## Data Availability

No data was used for the research described in the article.
